# Improving Surveillance of Human Tick-Borne Disease Risks: Spatial Analysis Using Multimodal Databases

**DOI:** 10.2196/43790

**Published:** 2023-08-23

**Authors:** Sarah P Maxwell, Chris Brooks, Dohyeong Kim, Connie L McNeely, Seonga Cho, Kevin C Thomas

**Affiliations:** 1 School of Economic, Political and Policy Sciences University of Texas at Dallas Richardson, TX United States; 2 Laboratory for Human Neurobiology Boston University School of Medicine Boston, MA United States; 3 Schar School of Policy and Government George Mason University Fairfax, VA United States; 4 Department of Geography University of California, Santa Barbara Santa Barbara, CA United States

**Keywords:** tick-borne disease surveillance, Lyme disease, tick bite encounter, One Health model, triangulation, entomology, entomological, tick, thematic mapping, spatial, risk, surveillance, vector

## Abstract

**Background:**

The extent of tick-borne disease (TBD) risk in the United States is generally unknown. Active surveillance using entomological measures, such as presence and density of infected nymphal *Ixodes scapularis* ticks, have served as indicators for assessing human risk, but results have been inconsistent and passive surveillance via public health systems suggests TBDs are underreported.

**Objective:**

Research using various data sources and collection methods (eg, Google Trends, apps, and tick bite encounters [TBEs] reports) has shown promise for assessing human TBD risk. In that vein, and engaging a One Health perspective, this study used multimodal databases, geographically overlaying patient survey data on TBEs and concomitant reports of TBDs with data drawn from other sources, such as canine serological reports, to glean insights and to determine and assess the use of various indicators as proxies for human TBD risk.

**Methods:**

This study used a mixed methods research strategy, relying on triangulation techniques and drawing on multiple data sources to provide insights into various aspects of human disease risk from TBEs and TBDs in the United States. A web-based survey was conducted over a 15-month period beginning in December 2020 to collect data on TBEs. To maximize the value of the covariate data, related analyses included TBE reports that occurred in the United States between January 1, 2000, and March 31, 2021. TBEs among patients diagnosed with Lyme disease were analyzed at the county level and compared to *I scapularis* and *I pacificus* tick presence, human cases identified by the Centers for Disease Control and Prevention (CDC), and canine serological data. Spatial analyses employed multilayer thematic mapping and other techniques.

**Results:**

After cleaning, survey results showed a total of 249 (75.7%) TBEs spread across 148 respondents (61.9% of all respondents, 81.7% of TBE-positive respondents); 144 (4.7%) counties in 30 states (60%) remained eligible for analysis, with an average of 1.68 (SD 1.00) and median of 1 (IQR 1) TBEs per respondent. Analysis revealed significant spatial matching at the county level among patient survey reports of TBEs and disease risk indicators from the CDC and other official sources. Thematic mapping results included one-for-one county-level matching of reported TBEs with at least 1 designated source of human disease risk (ie, positive canine serological tests, CDC-reported Lyme disease, or known tick presence).

**Conclusions:**

Use of triangulation methods to integrate patient data on TBE recall with established canine serological reports, tick presence, and official human TBD information offers more granular, county-level information regarding TBD risk to inform clinicians and public health officials. Such data may supplement public health sources to offer improved surveillance and provide bases for developing robust proxies for TBD risk among humans.

## Introduction

Tick-borne illness is steadily increasing across the United States. Lyme disease (LD), in particular, is the fastest growing vector-borne disease in the United States and accounts for the majority of all tick-borne diseases (TBDs) in the country [1,2]. Research using various data sources and collection methods (eg, Google trends, apps, and tick bite encounters’ [TBEs’] reports) has shown promise for assessing human TBD risk [3-9]. However, the extent of TBD risk is relatively unknown. Entomological measures, such as presence and density of infected nymphal *Ixodes scapularis* ticks, have served as indicators for assessing human LD risk [10-13], but results have been inconsistent [11,14]. These measures do not consider human behavior [11]. Creating models that incorporate human behavior and adverse events—for example, TBE recall—can produce improved and more nuanced approaches for assessing human TBD risk. Internet and database searches on LD have demonstrated that information-seeking behaviors share similar temporal and spatial trends with known epidemiological reports [8,9]. Similar information seeking behaviors among health care providers also follow similar regional and temporal patterns to know human disease trends [10].

LD is transmitted by ticks in the *Ixodes* genus. *I scapularis*, the blacklegged tick, and *I pacificus*, the western blacklegged tick, are the primary vectors of the spirochete (*Borrelia burgdorferi*) that cause LD [10,15]. The blacklegged tick transmits other pathogens, including *Anaplasma phagocytophilum*, *Borrelia miyamotoi*, *Babesia microti*, *Powassan virus*, and ehrlichiosis associated with *Ehrlichia muris* eauclairensis [10,16]. *I pacificus* is known to transmit *Borrelia burgdorferi* sensu stricto, *Borrelia mayonii*, *Anaplasma phagocytophilum*, and *Borrelia miyamotoi* [16]. In addition, transmission of TBD pathogens to humans can result in related coinfections, such as Babesia, ehrlichiosis, or granulocytic anaplasmosis [16,17]. Recent modeling suggests the expansion of both *I scapularis* and *I pacificus* in areas where surveillance previously did not indicate reported or established tick populations [18]. The spread of *I scapularis* and *I pacificus* ticks with accompanying pathogens points to LD and other TBDs as a major public health risk [10], but, again, the actual prevalence of multiple TBDs or coinfections from related tick encounters is largely unknown, posing a problem for public health planning. Public health systems face significant challenges in identifying the spread of tick populations, as well as the associated risk and burden of TBDs among humans [19]. TBDs in this regard—accounting for 95% of all vector-borne diseases in the United States—are an especially serious and growing public health threat, with LD accounting for more than 70% of cases [20].

Veterinary studies that capture TBDs are shown to be useful in identifying possible coinfections and providing an important basis for human risk comparison [11], especially in areas where the Centers for Disease Control and Prevention (CDC) indicates TBD existence but where the actual number of cases may be unknown [12]. This study incorporates multimodal data triangulation using TBE, canine serological reports, public health data, and tick presence indicators to assess human disease risk among survey respondents reporting a TBE and concomitant diagnosis of a TBD.

## Methods

### Study Design

Various studies have introduced the use of web-based and other survey data along with other sources of patient information, on matters such as TBEs to demonstrate promising approaches and indicators for investigating human disease risk [3,4]. This study builds upon and extends recent research to include data on human TBEs at the county level and associated self-reports of TBDs in not only officially designated endemic regions but, importantly, also regions perceived as nonendemic in the United States relative to known tick and human and canine disease case reports.

The analytical objective in this study was to assess human TBD risk by employing a mixed methods approach using triangulation techniques and multimodal databases, geographically overlaying TBE reports to official data sources. Specifically, a web-based survey was conducted over a 15-month period beginning in December 2020 to collect data on TBEs. To maximize the value of the covariate data, related analyses included only TBE reports that occurred in the United States between January 1, 2000, and March 31, 2021. Adopting a One Health perspective, TBEs among patients diagnosed with LD were analyzed at the county level and compared to *I scapularis* and *I pacificus* tick presence, human cases identified by the CDC, and canine serological data. Spatial analyses included multilayer thematic mapping and statistical analyses, involving the amalgamated use of county-level patient TBEs, canine serological cases, tick presence, official CDC-reported human cases as an indicator for human TBD risk, and US Census data. Mapping and analyses of data conducted at the county level across the resultant data sets were supplemented by Fisher’s exact tests of independence.

### Ethical Considerations

The study protocol was approved by the institutional review board at the University of Texas at Dallas (IRB-21-149). Institutional review board approval was obtained prior to the survey, under formal adoption of the Declaration of Helsinki. All answers were completely anonymous and patients consented to voluntarily take the survey. All survey respondents provided informed consent. No personally associated identification data were collected or reported as such. Human subjects were not compensated. Data are available upon request.

### Tick Bite Encounters and Respondent Data

The Tick Bite Encounter Survey (henceforth referred to as the “survey”) was conducted on the internet via social media and shared through selected national TBD-related nonprofit organizational websites. The survey was available beginning in December 2020 for 15 months via an anonymous link (administered by Qualtrics). Designed to engage individuals diagnosed with a TBD, participation was voluntary, with respondents constituting a convenience sample for the study. Survey respondents were asked to report their diagnoses, tick-bite recall by month and year, and by county or zip code and state where tick bites occurred. Respondents listed county or zip code where and when they recalled receiving up to 4 tick bites or TBEs, and data were assessed to ensure entries matched corresponding states as reported. To maximize the value of the covariate data (eg, CDC-positive LD diagnoses), only TBEs that occurred in the United States between January 1, 2000, and March 31, 2021, were included in the analysis. In addition, TBEs were only analyzed if the location provided by the respondent could be unambiguously localized to a single zip code or county. Selected demographic information was also collected, including those who did not recall a TBE.

Additional study end points derived from official national databases were obtained from two main sources: (1) official by-county databases maintained by the CDC and US Census Bureau: (A) total number of human LD cases that met CDC diagnostic criteria and were recorded by state health officials to the CDC between 2000 and 2019; (B) counties officially established and reported by the CDC to contain *I scapularis* or *I pacificus* ticks as of 2020). The CDC notes that “counties classified as ‘no records’ should not be interpreted as the tick being absent. No records could be a result of a lack of sampling efforts, tick collections, or a lack of reporting or publishing the results of sampling efforts” [21]. Accordingly, for each county, tick presence was coded as present, absent, or unknown (no data); and (C) county populations as reported by the US Census Bureau’s Intercensal Estimates for 2000 to 2010 and 2010 to 2020. (2) Companion Animal Parasite Council county-level databases: total number of serological tests conducted on canines in 2020 (specifically: number of tests positive for ehrlichiosis; number of tests positive for anaplasmosis; number of tests positive for LD). The maps were presented as demonstrating county-level overlap of TBEs by each human disease risk indicator (ie, canine serological reports, CDC LD cases, and tick presence). In addition to thematic and multilevel mapping analyses using the ESRI’s ArcGIS (version 10.7), cross tabulations are provided across all data sources for all counties containing 1 or more survey TBEs, aligning with the thematic maps.

## Results

### Overview

Statistical analyses were conducted at the county level for all counties which had at least 1 TBE reported on the survey. Of the 239 respondents who completed the web-based survey, 182 (76.2%) reported at least 1 TBE, as shown in Table 1. A combined total of 329 TBEs were reported, with an average of 1.82 (SD 1.11) and a median of 1 (IQR 1) TBE per TBE-positive respondent. TBEs were filtered to restrict the sample to respondents who met the following criteria: the TBE could be definitively localized to a single county or zip code in a US state, and the TBE could be definitively localized to a single year between 2000 and 2021, inclusive. Note that some respondents indicated the location of TBEs using zip codes that occasionally encompassed areas crossing and belonging to different counties; in those cases, the TBE was considered to have occurred in the zip code’s “primary county” [22] and was considered “definitively localized” for analytical purposes. After applying these criteria, a total of 249 (75.7%) TBEs were apparent across 148 respondents (61.9% of all respondents, 81.8% of “TBE-positive” respondents), 144 (4.8%) counties in 30 states (60%) remained eligible for analysis, with an average of 1.68 (SD 1.00) and median of 1 (IQR 1) TBEs per respondent. County-level statistics appear in Table 2, with 144 eligible counties and their county-level data in the final data set. Unless otherwise noted, all further county-level proportions and percentages described herein were derived from this set of 144 counties; likewise, all state-level statistics were derived from the set of 30 states. None of the 144 counties or 30 states analyzed was missing survey TBE data, as only counties with one or more reported TBEs were included in the analysis. Of these, 14 (9.72%) counties across 7 (23.33%) states reported no CDC LD cases. These data include official LD reports from the CDC; patient reports of TBEs via web-based survey; established tick presence, indicating counties with habitats suitable for ticks; and canine serological data for Lyme, ehrlichiosis, and anaplasmosis.

**Table 1:** Survey respondents and tick bite recall (December 2020 to March 2021).

		Recall tick bite, n (%)	Do not recall tick bite, n (%)	All respondents, n (%)
**Age**				
	9 to 45 years old	65 (66.3)	33 (33.7)	98 (41)
	46 or more years old	116 (82.9)	24 (17.1)	140 (58.6)
	Age not reported	1 (100)	0 (0)	1 (0.4)
	Total	182 (76.2)	57 (23.9)	239 (100)
**Number of years sick**				
	0 to 5 years	113 (77.4)	33 (22.6)	146 (61.1)
	6 or more years	67 (75.3)	22 (24.7)	89 (37.2)
	Not reported	2 (50)	2 (50)	4 (1.7)
	Total	182 (76.2)	57 (23.6)	239 (100)

**Table 2:** Number of US counties with reported TBDs^a^ by frequency of primary end points.

		Counties with disease indicator present, n (%)	Counties with no disease indicator present, n (%)	Data unavailable, n (%)	Total, n (%)
**Human cases**					
	CDC^b^ LD^c^	130 (90.3)	14 (9.7)	0 (0)	144 (100)
	Survey TBE^d^	144 (100)	0 (0)	0 (0)	144 (100)
**Tick presence**					
	*Ixodes scapularis*	110 (76.4)	34 (23.6)	0 (0)	144 (100)
	*I pacificus*	2 (1.4)	142 (98.6)	0 (0)	144 (100)
**Canine serological tests**					
	Canine any TBD	126 (87.5)	3 (2.1)	15 (10.4)	144 (100)
	Canine LD	113 (78.5)	16 (11.1)	15 (10.4)	144 (100)
	Canine anaplasmosis	106 (73.6)	23 (15.9)	15 (10.4)	144 (100)
	Canine ehrlichiosis	122 (84.7)	7 (4.9)	15 (10.4)	144 (100)

^a^TBD: tick-borne disease.

^b^CDC: Centers for Disease Control and Prevention.

^c^LD: Lyme disease.

^d^TBE: tick bite encounter.

### Self-Reported TBEs

**Figure 1:**
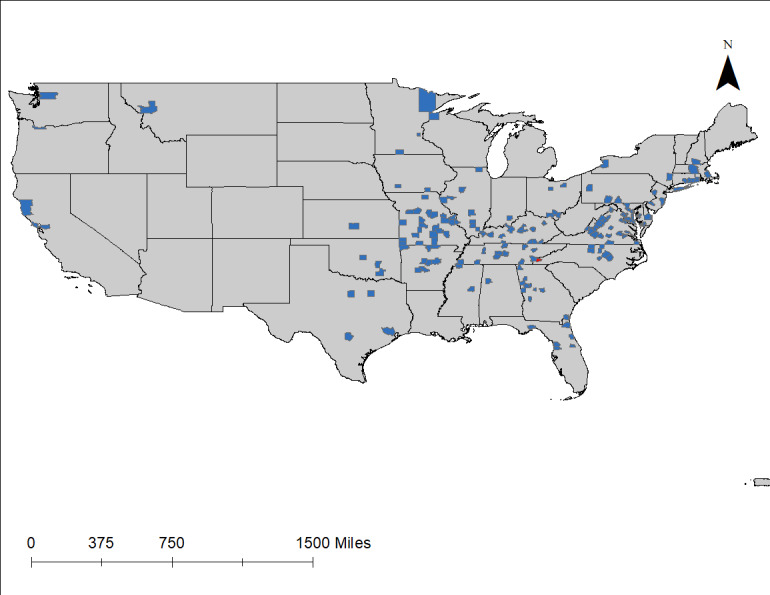
Survey respondents with LD and reported tick bite encounters by county. LD: Lyme disease.

**Figure 2:**
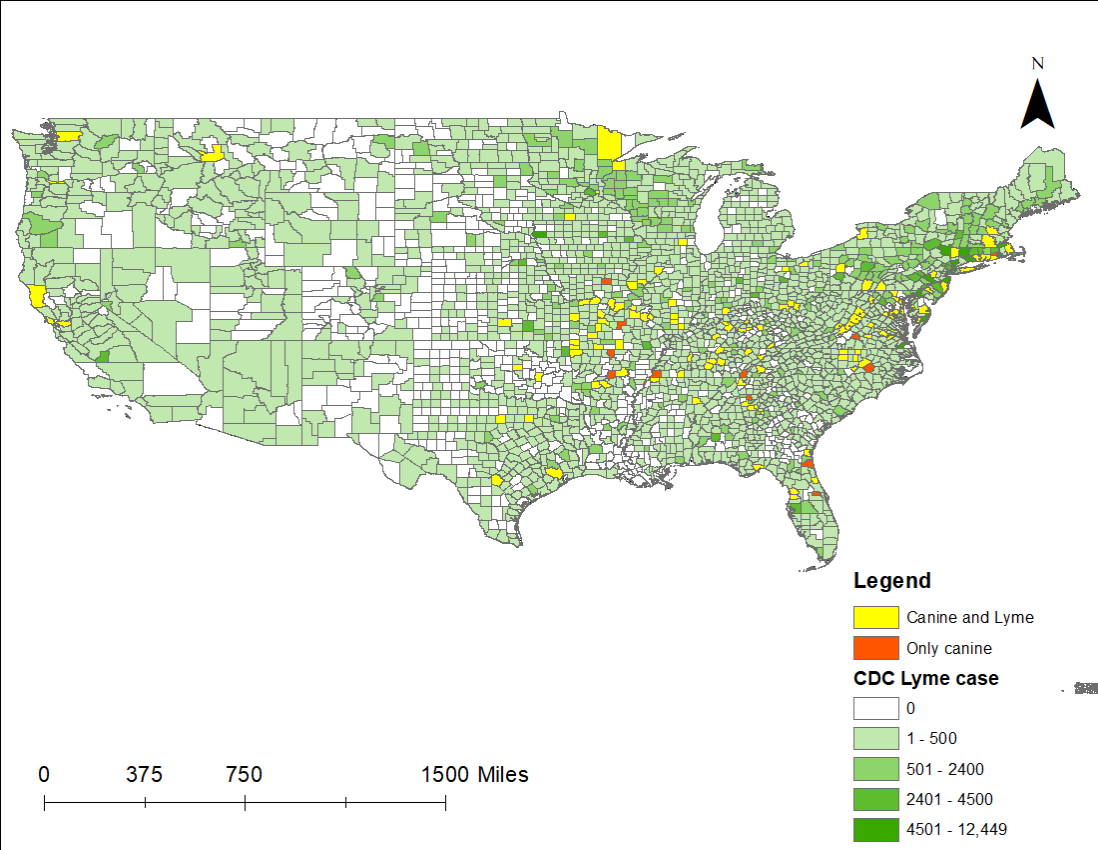
Respondent TBEs with canine and CDC LD cases by county (CDC LD cases 2000-2019). CDC: Centers for Disease Control and Prevention; LD: Lyme disease; TBE: tick bite encounter.

**Figure 3:**
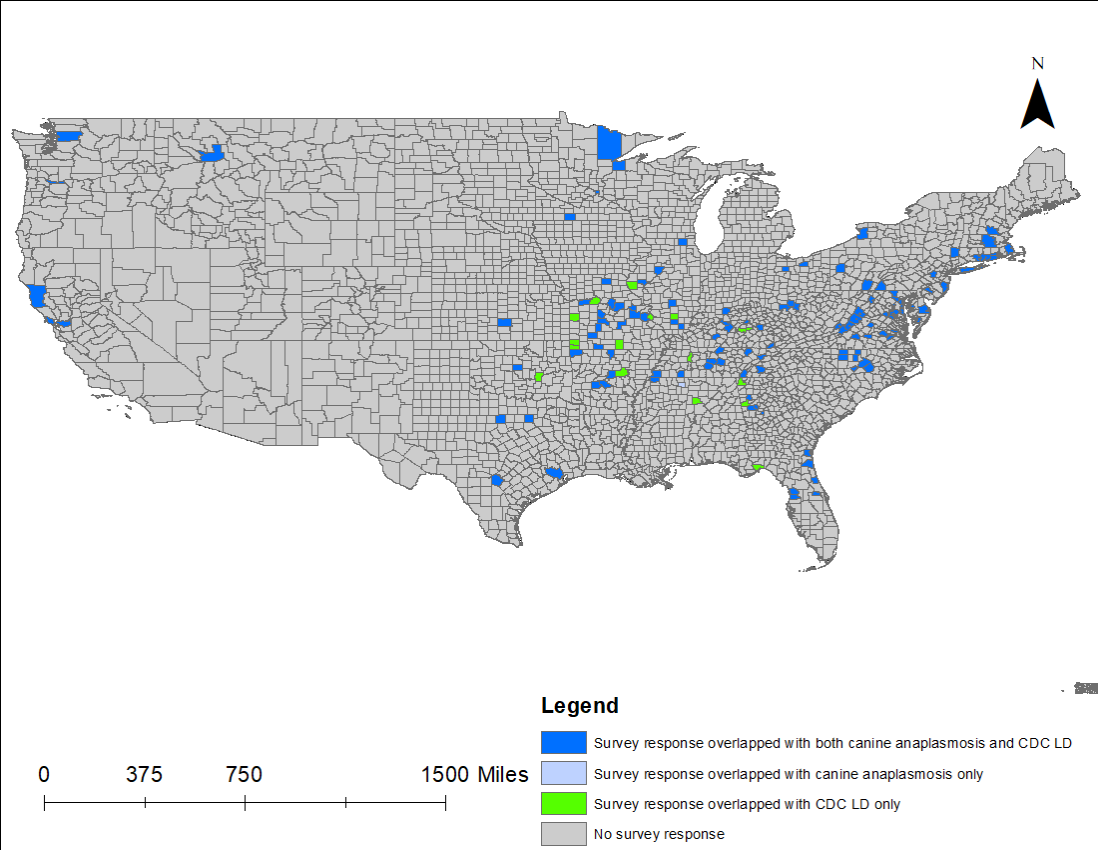
Respondent TBEs with canine anaplasmosis and CDC LD cases by county (CDC LD cases 2000-2019). CDC: Centers for Disease Control and Prevention; LD: Lyme disease; TBE: tick bite encounter.

**Figure 4:**
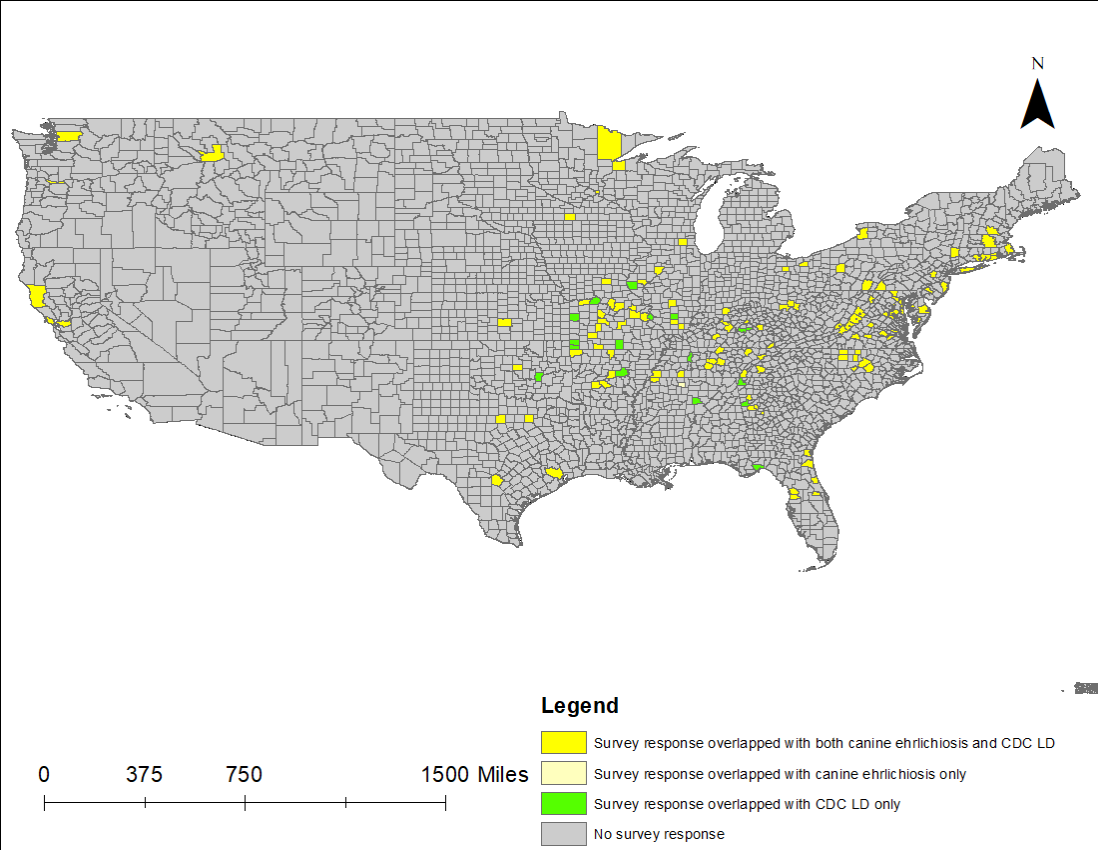
Respondent TBEs with canine ehrlichiosis and CDC LD cases by county (CDC LD cases 2000-2019). CDC: Centers for Disease Control and Prevention; LD: Lyme disease; TBE: tick bite encounter.

**Figure 5:**
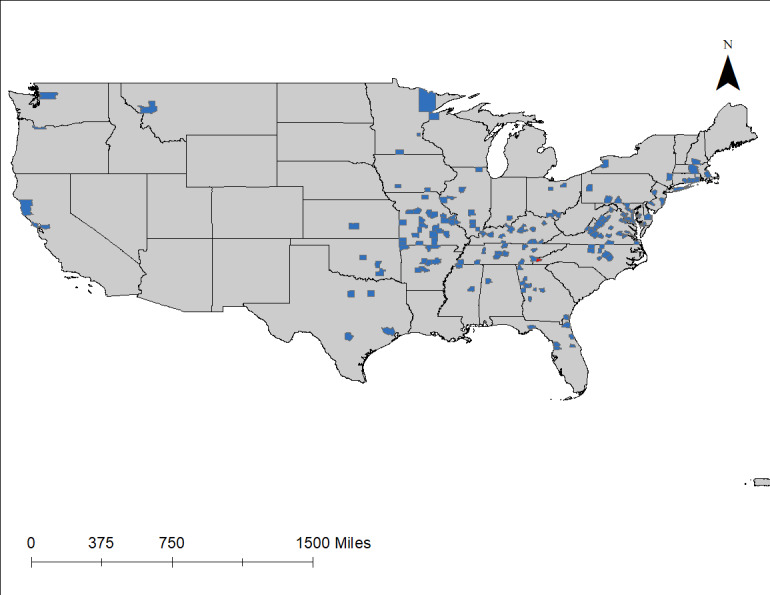
US counties with survey respondent reported TBEs and LD diagnoses, with TBEs matched to triangulated data points of tick presence or human or canine disease. LD: Lyme disease; TBE: tick bite encounter.

Using triangulation methods, Figures 1-5 show tick presence and the existence of human and canine disease by county in the United States. Multilayered thematic maps of self-reported TBEs among patients diagnosed with LD and coinfections, in addition to statistical alignments and comparisons of data regarding TBE reports and recall, was developed and performed. Taken together, the maps offer a representation of human TBD risk, based on the more nuanced approach engaging triangulation of human and canine reports in addition to tick presence. Survey-reported TBEs in 144 counties, as shown in Figure 1, are then compared to official disease and tick data. Official CDC data are also compared separately to demonstrate county level overlap via human and canine laboratory testing and tick presence.

Mapping and analyses of subsequent county-level data also included Fisher’s Exact tests of independence, as reported in Tables 3-6, to evaluate the likelihood that the co-occurrence or “county overlap” of given pairs of variables was random. A significant outcome (*P*<.05 level of significance) provides evidence that the 2 variables may be used as proxies for one other.

**Table 3:** Association across US counties by tick presence (significance set at *P*<.05).

Fisher exact test	*Ixodes pacificus* − Counties	*I pacificus +* Counties	Total
*I scapularis* – Counties	32	2	34
*I scapularis +* Counties	110	0	110
Total	142	2	144

**Table 4:** Association of US counties with survey respondent TBEs^a^ by canine LD^b^ and CDC^c^ LD cases (significance set at *P*<.05).

Fisher exact test	County has reports of CDC LD	County has no reports of CDC LD	Total
County has reports of canine LD	5	11	16
County has no reports of canine LD	5	108	113
Data unavailable	4	11	15
Total	14	130	144

^a^TBE: tick bite encounter.

^b^LD: Lyme disease.

^c^CDC: Centers for Disease Control and Prevention.

**Table 5:** Association of US counties by survey respondent TBEs^a^ based on canine anaplasmosis and CDC^b^ LD^c^ cases (significance set at *P*<.05).

Fisher exact test	County has reports of CDC LD	No county reports of CDC LD	Total
County has reports of canine anaplasmosis	5	18	23
County has no reports of canine anaplasmosis	5	101	106
Data unavailable	4	11	15
Total	14	130	144

^a^TBE: tick bite encounter.

^b^CDC: Centers for Disease Control and Prevention.

^c^LD: Lyme disease.

**Table 6:** Association of counties by survey respondent TBE^a^ with canine ehrlichiosis and CDC^b^ LD^c^ Cases (significance set at *P*<.05).

Fisher exact test	County has CDC reports of LD	County has CDC reports of no LD	Total
County has reports of canine ehrlichiosis	3	4	7
County has reports of no canine ehrlichiosis	7	115	122
Data unavailable	4	11	15
Total	14	130	144
Fisher exact outcome	<0.05	<0.05	<0.05

^a^TBE: tick bite encounter.

^b^CDC: Centers for Disease Control and Prevention.

^c^LD: Lyme disease.

### Human LD, Survey TBEs, and Canine LD

Figures 2-5 present integrated, multilayer thematic maps that show overlap of survey respondent TBEs with canine cases of LD, ehrlichiosis, and anaplasmosis, and human cases of LD (the only available county-level TBD data from the CDC). Figure 2 presents a thematic overlap of CDC and canine cases of LD within counties with at least 1 survey-reported TBE. Yellow counties indicate containing canine LD, CDC-reported LD cases, and where TBE survey respondents reported a TBE and concomitant disease. Red counties represent overlap among canine LD and TBE survey respondents, but without CDC reports of human LD. Green graduated shaded counties are all LD counties in the United States as reported by the CDC. Table 4 provides statistical data representing LD by county for respondent TBE, canine LD, and CDC LD. Some counties do not have canine data, so disease presence among dogs in those counties is unknown.

Figure 3 offers a map of CDC data and canine cases of anaplasmosis within TBE survey respondent counties. Blue counties indicate where canine cases overlap with CDC LD cases and survey-reported TBEs. Light blue counties represent overlap among canine LD and TBE survey respondents, but without CDC reports of human LD. Green counties are all LD counties with TBE survey respondent overlap. Table 5 highlights the thematic mapping overlaying, indicating significant association between canine anaplasmosis and CDC cases of LD within counties that had reports of a human TBE.

Figure 4 presents a thematic overlay of CDC and canine ehrlichiosis cases within TBE survey respondent counties. Yellow counties indicate where canine LD cases overlap with CDC LD cases and survey reported TBEs. Light yellow shading represents overlap between canine LD and TBE survey respondents, but without CDC reports of human LD. Green shading likewise represents counties with overlap between CDC LD and TBE survey data, but with no canine LD cases. Table 6 highlights the thematic mapping indicating significant associations between canine ehrlichiosis and CDC reported cases of LD within counties with human TBE reports.

In sum, the maps offer visual representation of TBE overlap with official data from 3 primary sources: tick presence, CDC-reported LD cases, and positive canine tests for LD, ehrlichiosis, and anaplasmosis. Using thematic overlays to triangulate matching human disease risk, every county overwhelmingly matched TBE survey reports and at least 1 disease risk indicator from official sources. Figure 5 is a summary map, depicting all counties where a survey respondent reported a TBE and a LD diagnosis and where the TBE matched at least one of the triangulated data points of tick presence or human or canine disease. The scope of influence is substantial in this respect; out of a total of 144 counties included in the final analytical data set, only 1 did not overlap with an official source of disease risk—Graham County, North Carolina—which appears as the only county in red on the summary map in Figure 5. In other words, the respondent TBEs and subsequent self-reported LD offer a one-to-one match with human or canine official serological reports. The lack of overlap in Graham County does not mean that disease risk does not exist but, rather, was the result of missing canine data. In fact, neighboring counties showed positive serological tests for LD, ehrlichiosis, and anaplasmosis in canines, and ticks do not adhere to county lines.

## Discussion

### Implications of the Relative Proportions of CDC LD Cases and Survey TBEs

To date, surveillance of TBDs typically is presented in aggregate national and state level databases, hindering analyses at levels that may be more revealing and provide more detailed depictions of relevant indications of TBD risk. The current public health system in the United States does not recognize as endemic areas that may cluster in various states outside of the Northeast. Data for most TBDs are provided by the CDC, aggregated from state public health departments following positive laboratory results. Although county-level data on TBDs are collected by some states, the CDC reports such data for LD only. In addition, while LD reports are available by county, no distinctions are drawn between locally as opposed to travel-acquired disease. Accordingly, TBE likelihood—which is geographically dependent—could not be confidently determined from such data alone.

However, multilayer thematic mapping has demonstrated clear overlapping among TBEs and concomitant human disease reports with canine and CDC LD reports and tick presence data by county. As noted, previous studies have demonstrated the utility of multilayer thematic mapping using canine and self-reported TBEs, particularly as it pertains to ecologically distinct regions [23]. Specifically, the survey sample, although relatively small, was national in scope. However, respondents reported TBEs only in western, mid-Atlantic, and eastern regions where LD and ticks are either established or considered endemic. Note that this could be an artifact and limitation of the data with the use of respondents as a convenience sample. In any case, a one-to-one match was found for TBE and human disease self-reports with at least one of the human disease risk indicators. The critical point here, as revealed through the mapping and the statistical analyses, is the substantial overlap in the TBE counties among canine (especially canine ehrlichiosis), CDC reported LD cases, and presence of ticks carrying associated pathogens.

### Triangulating TBD Risk via Proxy Data

Developing robust proxies for human TBD risk to address data and testing limitations is an important analytical undertaking, especially until objective, standardized, and centralized diagnostic monitoring can be implemented for a more detailed picture below the state level as needed. Research has pointed to similar spatial distributions and county-level findings among canines and humans using official public health data, in addition to self-reports of TBEs and subsequent diagnoses (both clinical and CDC+) and tick infectivity [23-26]. The use of other data sources, including patient surveys, also points to other surveillance data and techniques that have the potential to inform public health practitioners when local data are scarce or clinical evaluations are made in geographic areas not generally considered endemic [27,28].

This study has explored paths for improving disease surveillance by triangulating TBD risk via proxy data. Proxies were considered to help establish risk, relying on “indirect data” such as surveys and veterinary assessments to supplement and extend official data when available. The use of canine TBDs as proxies was supported by research indicating, for example, links between companion pets and human disease. In fact, owners of dogs and cats are at increased risk of encountering ticks and of developing TBDs [29], which also reflects the One Health perspective given connections among humans and their pets. In other words, canine TBD data are not fully divested from human TBD data and, so, with proper consideration of their differences and precise characterization of their common factors, they can usefully serve as proxies, as demonstrated in this study.

### Limitations and Recommendations for Future Work

The survey was intentionally aimed at individuals likely to have experienced TBEs and to have a high probability of participation and response (eg, web-based support groups focused on TBDs). This approach naturally sat the survey respondent sample apart from the general population and could lead to underestimation of TBEs. In addition to survey limitations and possible bias and simple recollection errors by respondents, some statistical issues can also be noted. In particular, the canine data represented cases only from 2020, rather than across the 2000-2021 study period.

Future work will incorporate more nuanced multilevel models that can account for unequal variances, mixed and fixed effects, nesting, and other confounding issues. Additional data too will address broader contextual concerns and data set gaps. For example, other tick species (eg, the Lone Star Tick) can act as vectors for TBDs and are extant in different geographical locations. Inclusion of such entomological data can improve analytical coverage. In addition, expanded historical data will help elucidate long-term trends that may not be detectable over shorter periods and will aid in outcome interpretation.

### Conclusions

Controversy arises as a burgeoning population reports TBD diagnoses that do not match official reports. LD and other TBDs can be difficult to diagnose and physicians and health care professionals may be unaware of growing risks and public health threats in locally clustered areas, particularly in areas generally considered nonendemic [23]. Diagnostic ambiguity is exacerbated by symptomatic overlap in TBD presentation, as well as insufficient tracking and characterization of the various vectors and TBEs that cause them.

To date, surveillance of TBDs in the United States is a patchwork of activities that are unable to provide a satisfactory picture of risk or infection rates across TBDs, not to mention overlapping comorbidities in infected humans. Resulting controversies plague the patient-physician-public health relationship and discourse, with LD and other TBD sufferers turning to advocacy in demand for diagnosis and treatment. The importance of identifying opportunities to improve surveillance and data for determining TBD risk cannot be overstated for informing the public and the medical community.

## Abbreviations

CDC: Centers for Disease Control and Prevention
LD: Lyme disease
TBD: tick-borne disease
TBE: tick bite encounter

## Data Availability

The data that support the findings of this study are available from the corresponding author upon reasonable request.
